# On the Secretion of Sugar and Bile in the Liver

**Published:** 1856-03

**Authors:** J. Moleschott


*On the Secretion of Sugar and Bile in the Liver.* By Dr. J. Moleschott.

In the Comptes Rendus of the French Institute, (May, 1855,) we find a communication from the eminent physiologist of Heidelberg, Professor Moleschott, designed to report a series of experiments which he published in 1852, but which are not generally known, which tend to prove that the liver is not an excretory organ.

Professor Moleschott has succeeded, in a large number of cases, after extirpating the livers of frogs, in keeping these animals alive for two or three weeks after the operation. It is well known that after the kidneys are removed, urea accumulates in the blood. Now, if the liver is only an apparatus of filtration, we should expect to find organic acids, coloring matter of bile, and sugar, in the blood or tissues of animals from which this viscus has been removed. Dr. Moleschott examined the blood, muscles, gastric juice, and urine of frogs from which the liver had been removed from one to twenty-one days, repeating and varying his analyses. He could not detect a trace of sugar, or of the immediate element of bile.

He concludes that sugar and bile are formed in the liver. For the first fact, science is indebted to M. Claude Bernard. In regard to bile, Muller first proved that it was produced by the liver. Kunde and Lehmann corroborated his experiments. But these savants preserved life in the animals in which they extirpated the liver for only two or three days.

Frogs from which the liver has been removed exhale far less carbonic acid than the same animals when intact. Dr. Moleschott has compared frogs without livers with frogs from which he had removed the legs, in order that they should lose as much blood as in excision of the liver, and also with intact animals. All the frogs were from the same marshes, of the same sex, and as nearly as possible of the same size. The experiments were made the same day, in a room of equable temperature, and under the same atmospheric pressure. Twenty-six experiments were made for each category. One hundred grammes of unmutilated frogs exhaled in twenty-four hours 0.566 of carbonic acid; 100 grammes of frogs of which the legs had been amputated, gave 0.457; and the same weight of frogs without livers, gave 0.332. These figures give the average or mean result of the numerous experiments. These facts prove that the removal of the liver diminishes the elimination of carbonic acid in a degree altogether beyond what might be explained by the loss of blood inevitble in such an operation.—*Virginia Medical Journal.*

